# Endogenous lentivirus in Malayan colugo (*Galeopterus variegatus*), a close relative of primates

**DOI:** 10.1186/s12977-014-0084-x

**Published:** 2014-10-04

**Authors:** Tomáš Hron, Helena Fábryová, Jan Pačes, Daniel Elleder

**Affiliations:** Institute of Molecular Genetics, Academy of Sciences of the Czech Republic, Vídeňská 1083, 14220 Prague, Czech Republic

**Keywords:** Endogenous lentiviruses, Dermoptera, Paleovirology

## Abstract

**Background:**

A significant fraction of mammalian genomes is composed of endogenous retroviral (ERV) sequences that are formed by germline infiltration of various retroviruses. In contrast to other retroviral genera, lentiviruses only rarely form ERV copies. We performed a computational search aimed at identification of novel endogenous lentiviruses in vertebrate genomes.

**Findings:**

Using the *in silico* strategy, we have screened 104 publicly available vertebrate genomes for the presence of endogenous lentivirus sequences. In addition to the previously described cases, the search revealed the presence of endogenous lentivirus in the genome of Malayan colugo (*Galeopterus variegatus*). At least three complete copies of this virus, denoted ELVgv, were detected in the colugo genome, and approximately one hundred solo LTR sequences. The assembled consensus sequence of ELVgv had typical lentivirus genome organization including three predicted accessory genes. Phylogenetic analysis placed this virus as a distinct subgroup within the lentivirus genus. The time of insertion into the dermopteran lineage was estimated to be more than thirteen million years ago.

**Conclusions:**

We report the discovery of the first endogenous lentivirus in the mammalian order Dermoptera, which is a taxon close to the Primates. Lentiviruses have infiltrated the mammalian germline several times across millions of years. The colugo virus described here represents possibly the oldest documented endogenization event and its discovery can lead to new insights into lentivirus evolution. This is also the first report of an endogenous lentivirus in an Asian mammal, indicating a long-term presence of this retrovirus family in Asian continent.

**Electronic supplementary material:**

The online version of this article (doi:10.1186/s12977-014-0084-x) contains supplementary material, which is available to authorized users.

## Findings

The lentiviruses have been described in several mammalian orders, including Primates, Artiodactyls, Perissodactyls, and Carnivores. They are the cause of a variety of chronic diseases and constitute a major public health concern, especially due to the HIV/AIDS pandemic. In contrast to other retroviral genera, lentiviruses rarely generate ERV copies [1]. The ERVs are formed following germline infection and further vertical transmission of the integrated provirus [2]. The presence of such genomic “viral fossils” enables the study of long-term evolutionary history and evolution of lentiviruses [1]. The first endogenous lentivirus has been described in 2007 in the genome of European rabbit [3]. Since then, there have been only a few additional reports of lentiviruses infiltrating into the genomes of hares, lemurs and ferrets [4-8]. We have performed a large-scale screening of all publicly available vertebrate genomes for the presence of endogenous lentivirus sequences. Here, we report the identification of the first endogenous lentivirus in the mammalian order Dermoptera, in the genome of the Malayan colugo (*G. variegatus*). We discuss the genomic and phylogenetic characteristics of this virus, which place it as one of the oldest described members of the lentivirus genus.

**Figure 1:**
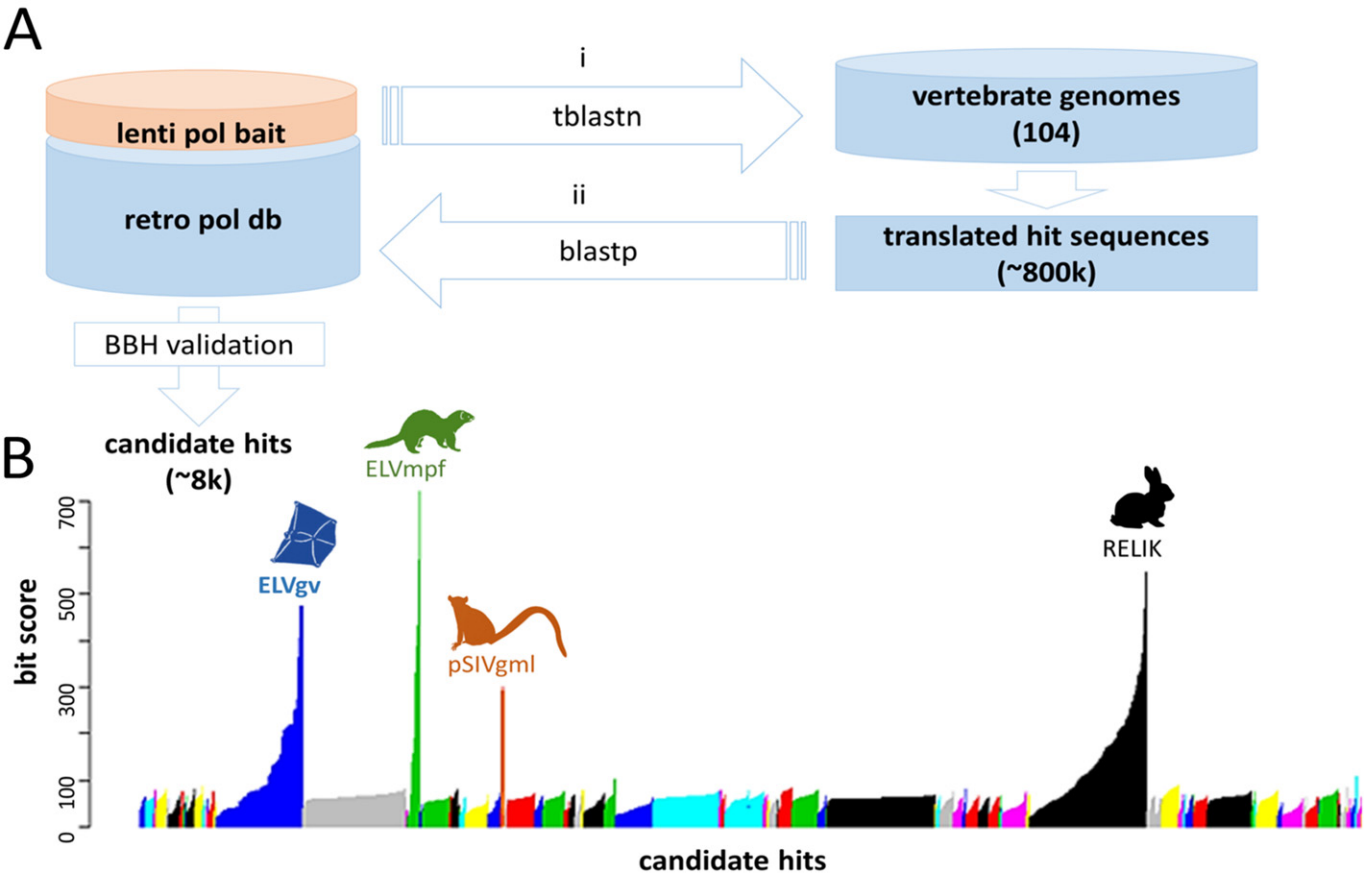
**Screening for lentiviral ERVs. (A)** Schematic depiction of the computational screening pipeline. The first step of the best bidirectional hit (BBH) strategy was performed by tblastn search in vertebrate genome database to identify candidate endogenous lentivirus fragments (i). In this step the following Pol amino acid sequences were used as baits: human immunodeficiency virus type 1 (HIV-1), feline immunodeficiency virus (FIV), Visna/maedi virus, rabbit endogenous lentivirus type K (RELIK), gray mouse lemur prosimian immunodeficiency virus (pSIVgml), and domestic ferret (*Mustela putorius furo*) endogenous lentivirus (ELVmpf). The cutoff for the blast search was set at E-value < 10^−5^. To filter out non-lentiviral sequences, translated hits were used as a query for backward blastp search against database of retroviral Pol sequences belonging to all retroviral genera (ii). Hits aligned with the best bit score to lentiviral sequences in the backward blast search were further analyzed. **(B)**. Graph shows bit scores of all lentiviral candidate hits ordered by species in which they were found. Each species is represented by different color. Newly discovered lentiviral sequence in colugo (ELVgv) as well endogenous lentiviruses in rabbit [3], domestic ferret [6], and gray mouse lemur [4,5] are indicated. Previously published endogenous lentivirus sequences were excluded as baits for their corresponding host species (e.g. RELIK against the rabbit genome) to avoid identical matching of the hits.

We have implemented a computational approach based on automated BLAST searches and the best bidirectional hit (BBH) strategy against custom retroviral database. This enabled us to screen for candidate lentiviral sequences in multiple genomic datasets (Figure 1A). A search of 104 publicly available vertebrate genomes recovered 8,179 candidate hits, each aligned to lentiviral sequence with a given bit score (Figure 1B). We identified false positive bit scores <100 in majority of animals. However, a few hits from rabbit, domestic ferret, and grey mouse lemur reached significantly higher bit scores. These sequences corresponded to previously described endogenous lentiviruses in the above mentioned species [3-6,8]. High scoring hits were also found in the genome of colugo. The matching sequences were manually extracted and found to cluster robustly with lentiviruses upon preliminary phylogenetic analysis. The endogenous lentivirus in the *G. variegatus* genome was denoted ELVgv.

Further BLAST searches of the colugo genomic contigs revealed the presence of three complete ELVgv proviruses (provirus *Ι* at positions 11,594-19,841 of contig JMZW01084956; provirus *ΙΙ* at positions 14,164-23,469 of contig JMZW01174031; provirus *ΙΙΙ* at positions 40,701-51,516 of contig JMZW01021293). This search also identified approximately 100 solo long terminal repeats (LTR), which are formed by recombination between the two LTRs flanking the viral internal sequences [9]. The BLASTn parameters employed for the identification of solo LTRs were the following: e-value < 10^−100^, identity to the LTR of full-length ELVgv provirus at least 80%, and coverage at least 50%. In addition, several smaller contigs containing fragments of internal virus sequences were detected (data not shown). The colugo genome assembly covers majority of the genome (assembly size 2.8 Gbp, accession number JMZW00000000), therefore it can be assumed that there are at least three complete provirus copies and ~30 times more solo LTRs per genome.

**Figure 2:**
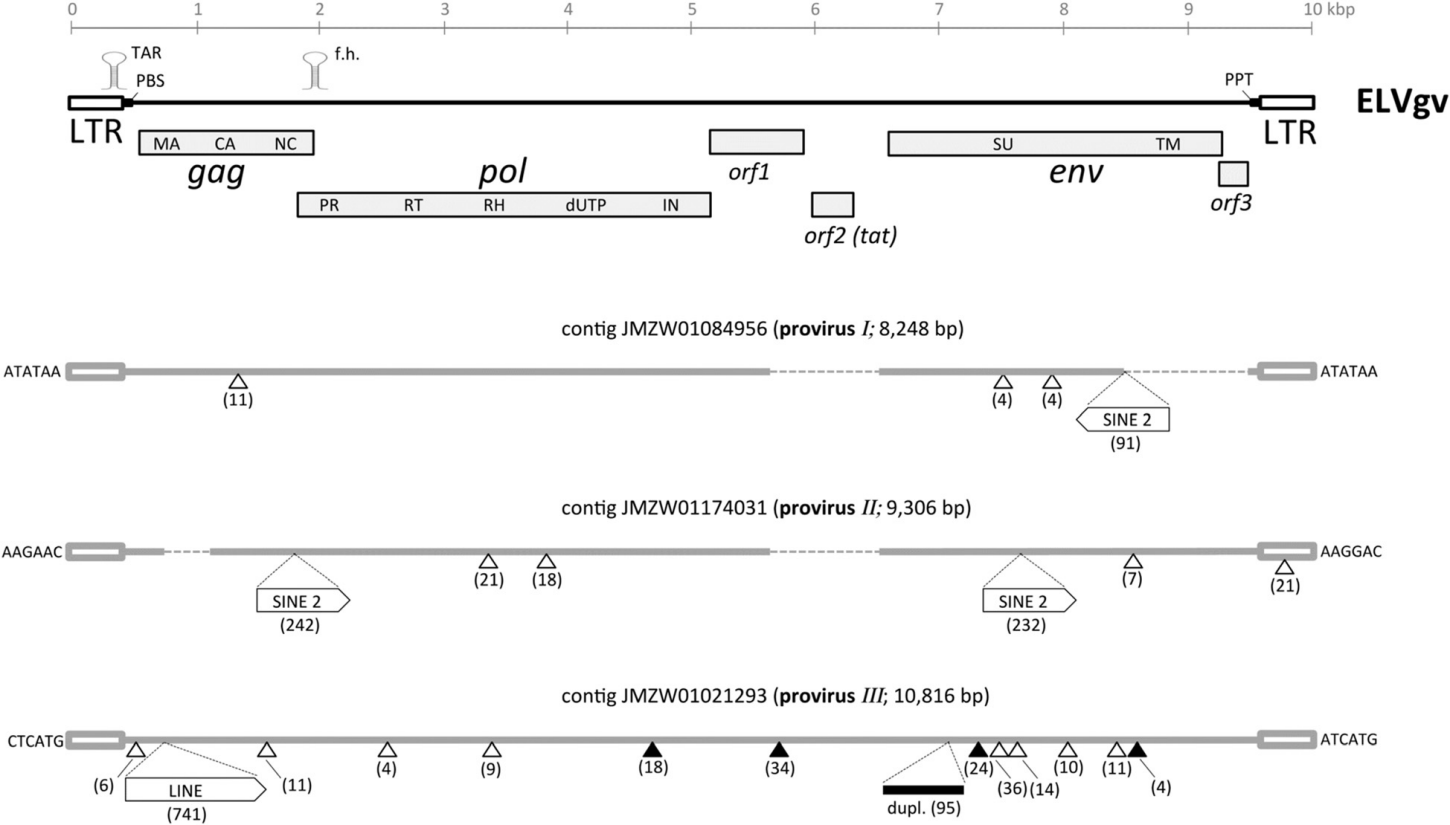
**Genome organization of ELVgv.** The consensus sequence of ELVgv is shown schematically below the scale. The position of open reading frames and other genomic features is indicated. The structure of the three complete proviruses recovered from the whole genome shotgun (WGS) contigs is shown below; their accession numbers are [GenBank:JMZW01084956], [Genbank:JMZW01174031], and [Genbank:JMZW01021293]. For each contig, the length of the provirus and its corresponding target site duplication is shown. Deletions and insertions >3 bp are depicted by open and closed triangles, respectively, together with their length. The four longest deletions are indicated by thin dashed line. Insertions of retroelements were detected by Censor [11] and are depicted by large open arrows, drawn not to scale. ENV coding regions were characterized with the help of specialized prediction servers [12-14]. LTR, long terminal repeat; MA, matrix; CA, capsid; NC, nucleocapsid; PR, protease; RT, reverse transcriptase; RH, RNaseH; dUTP, dUTPase; IN, integrase; PBS, primer binding site; f.h., frameshift hairpin; PPT, polypurine tract; SINE, short interspersed nuclear element; LINE, long interspersed nuclear element; dupl., duplication.

Alignment of all available contig sequences was used to reconstruct the ELVgv full consensus sequence (Figure 2 and Additional files 1 and 2). The reconstructed provirus is 10,040 bp long and flanked by LTRs of approximately 420 bp. The genome organization is typical for a lentivirus, with three long open reading frames (ORFs) corresponding to *gag*, *pol*, and *env* genes. The *gag* and *pol* genes lie in different reading frames and *pol* is predicted to be translated via ribosomal frameshifting. Consistent with that, a hairpin RNA secondary structure is predicted in the *gag*-*pol* overlapping region (Additional file 3) [10]. A feature present also in other nonprimate lentiviruses is the occurrence of dUTPase between RNaseH and integrase domains of the ELVgv *pol* gene. Two short ORFs, named *orf1* and *orf2*, were detected in the intervening region between *pol* and *env* (Figure 2 and Additional file 1). The *orf2* (103 aa) could be identified by sequence similarity as a *tat* gene (Additional file 3). A corresponding TAR (transactivating responsive region) was predicted in the LTR downstream of the putative promoter (Additional file 3) [10]. The *orf1* gene (272 aa) partially overlaps in an alternative reading frame with *pol*. No sequence similarity of *orf1* with any lentiviral accessory gene was detected. According to its size and genomic location, *orf1* might encode a *vif* homolog. A third short ORF, *orf3* (83aa), partially overlaps with the end of *env*, and extends towards the 3’LTR. As for *orf1*, the sequence of *orf3* did not point to any specific accessory gene. The location and size indicate that *orf3* might be a homolog of lentiviral *rev*. The presence of a limited number of viral accessory genes is in agreement with the previously described evolutionarily ancient lentiviruses [4].

**Figure 3:**
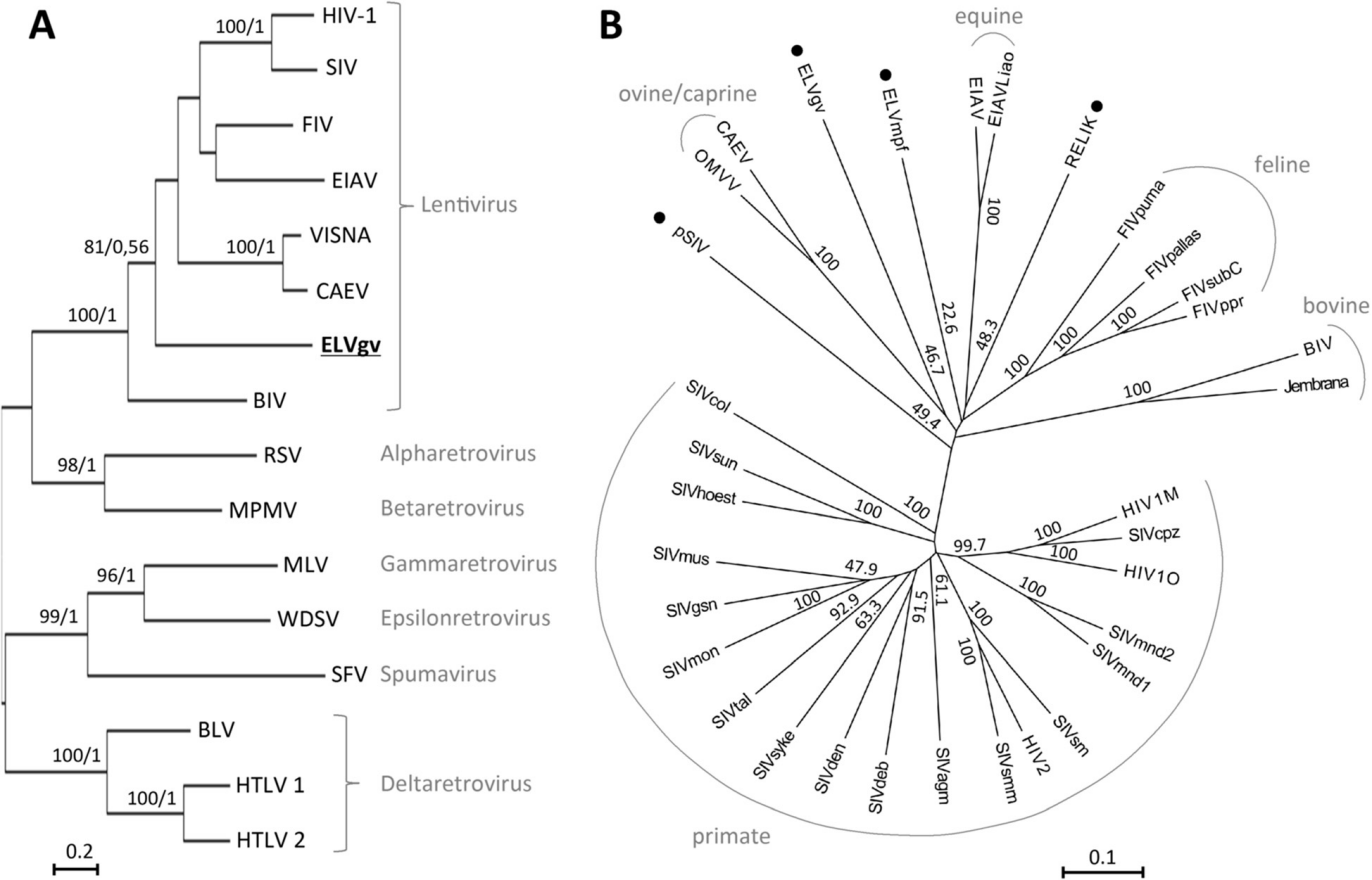
**Phylogenetic relationship of ELVgv to other retroviruses. (A)** Phylogeny of ELVgv and other retroviruses, based on alignment of RT amino acid sequences (Additional file 4 contains the alignment in FASTA format and the full names of the retroviruses). The alignment was generated in MEGA5 software [15] using the MUSCLE algorithm [16]. The ML tree was constructed in MEGA5 software, using the rtREV amino acid substitution matrix [17], Nearest-Neighbor-Interchange ML heuristic method and otherwise default parameters. Support for ML tree was assessed by 1,000 nonparametric bootstrap replicates. Bayesian analysis was run for 200,000 steps, sampling every 1,000 steps and discarding first 25% of the trees. Average standard deviation of split frequencies converged during 10,000 steps bellow 0.001. The amino acid model F81 in program MrBayes was used [18]. The support values are indicated above the branches (percent Bootstrap scores/Bayesian posterior probabilities). The ELVgv position is highlighted in bold and underlined. The names of the retrovirus genera are shown on the right. **(B)** Phylogenetic relationship of ELVgv to other exogenous and endogenous lentiviruses. The analysis was based on alignment including 2,350 most conserved nucleotides of *gag-pol* from 31 lentiviruses (used also in Gilbert *et al*. [5]), together with ELVmpf [6,8], and ELVgv from this work (alignment available in Additional file 5). The alignment was generated in MEGA5 program [15] using the MUSCLE algorithm [16]. The ML analysis was performed using MEGA5 program under Tamura-Nei model, Nearest-Neighbor-Interchange ML heuristic method and otherwise default parameters. Bootstrap supports (percent out of 1,000 replicates) are shown. Grey lines designate groups of exogenous lentiviruses. The endogenous lentiviruses are denoted by black dots. Scale bars indicate number of substitutions (amino acid or nucleotide) per site.

To establish the phylogenetic placement of ELVgv within lentiviruses, we have aligned the amino acid sequence of the highly conserved reverse transcriptase (RT) region of *pol* with sequences from representatives of all retrovirus genera. In subsequent phylogenetic analysis using both maximum likelihood (ML) and Bayesian methods, ELVgv RT clustered inside the lentivirus clade with high support (ML bootstrap 100, Bayesian posterior probability = 1) (Figure 3A; alignment is available in Additional file 4). In accordance with this clustering, the highest-scoring BLASTp hits of ELVgv *gag*, *pol* and *env* genes were the genes from a lentivirus, feline immunodeficiency virus (FIV; the similarity/identity to FIV counterparts of *gag*, *pol* and *env* genes were 48%/31%, 54%/35% and 27%/17%, respectively). To analyze the relationship of ELVgv to other lentiviruses in more detail, we have used the dataset of conserved regions of *gag* and *pol* lentiviral sequences from Gilbert *et al*. [5], together with the recently described ELVmpf [6,8]. ML phylogenies generated using this alignment placed ELVgv as a deep branch of the lentivirus tree (Figure 3B; alignment is available in Additional file 5), forming a distinct lentivirus subgroup. As in previous analyses of lentivirus phylogenies, basal nodes did not have strong support [3-6], and the ML tree differed slightly from the phylogeny obtained by Bayesian analysis (compare Figure 3B and Additional file 6). While in the ML analysis ELVgv clustered weakly (bootstrap support 46.7) together with the ovine/caprine lentivirus subgroup, in the Bayesian tree it formed an isolated deep branch. Separate analysis of the *gag* and *pol* genes excluded any evident recombination event (data not shown). Re-running the analysis with the three individual provirus sequences instead of the reconstructed ELVgv consensus sequence also did not influence the results (ML tree in Additional file 7). Therefore, the precise relationship of ELVgv to primate and nonprimate lentivirus groups could not be determined.

There are four lines of evidence suggesting that ELVgv inserted into the colugo germline millions of years ago. First, the three complete proviruses accumulated many genetic defects. These include insertions and deletions of various sizes, multiple frameshifts and stop codons, and insertions of SINE and LINE sequences (Figure 2). Second, the solo LTRs are formed only after prolonged existence in the germline [9]. Third, comparison of LTR sequences belonging to individual proviruses can be used to estimate the insertion times [19]. These estimates are only very approximate and use the fact that the 5’ and 3’ LTRs are identical at the time of insertion. Any divergence between them is supposed to have been formed postintegration and at neutral substitution rate of the host genome [19]. We assumed the range of mammalian substitution rates to be between 2.2 and 4.5 × 10^−9^ per site per year [20,21]. The provirus *Ι* had 20 differences between 5’ and 3’ LTRs, resulting in an estimated time of insertion of 5.1 - 10.3 million years ago (MYA). Similarly, proviruses *ΙΙ* and *ΙΙΙ* yielded integration time estimates of 10.1 - 20.7 MYA and 13.2 - 27.0 MYA, respectively. We note that all three proviruses have different perfect or almost perfect target site duplications, indicating that they have not undergone recombination events after integration and that the LTRs belong to the original integrating virus (Figure 2). The genetic distances between the individual proviruses are between 0.078 and 0.105 substitutions per site. However, we did not attempt to use the distances to estimate the integration age. It is not known whether they were formed by independent insertions of circulating exogenous virus, by reinfection of germline cells or by intracellular retrotranspositions. In addition, the assembly of genomic contigs from short Illumina reads is inherently very difficult in repeat regions that include ERVs. Especially the parsing of reads among the orthologous internal positions of different proviruses might not be exact. A fourth line of evidence pointing to ancient origin of ELVgv came from the fact that seven of the solo LTR insertions reside in regions of apparent segmental genomic duplications (Additional file 8). The virus integration must have happened before the duplication event. This allows estimating the lower age limit of the integrations, which is up to 7 MYA.

The Malayan colugo (*G. variegatus*) belongs to a tiny order Dermoptera, which contains only one additional extant species, Philippine colugo (*Cynocephalus volans*) [22]. Colugos, primates, and treeshrews (Scandentia) cluster together in a taxonomic subgroup Euarchonta [23]. There is an ongoing dispute about the placement of Dermoptera. Chromosome painting comparison of these groups suggested that tree-shrews and colugos had a closer phylogenetic relationship and formed a sister group to primates [24]. However, screening of protein-coding exons indicated that colugos are closer to primates than to tree-shrews [25]. In either scenario, the split of the dermopteran lineage is estimated to be between 80–90 MYA. This is considerably older than the highest estimate of the ELVgv insertion age and indicates that the genome invasion was an independent event in Dermoptera. In accordance with this fact, about half of the ELVgv integration sites could be identified in primates and other mammals in its empty pre-integration form (data not shown). It will be informative to ascertain the presence of ELVgv in the *Cynocephalus* genus, which diverged from the genus *Galeopterus* about 18.3 MYA [25,26], and in the multiple subspecies of *Galeopterus variegatus* [22]. The timescale of the ELVgv genome infiltration is at the upper limit of the previously described lentiviral invasions in leporid species (12 MYA) [3,7], lemurs (4.2 MYA) [4,5] and ferrets (12 MYA) [6,8]. The source and ancestral relationships between these ancient lentiviruses are not possible to resolve with the current data due to the inconclusive nature of phylogenetic analyses. The ancient origin and presence in a potentially closest relative of primates makes the colugo virus an interesting addition to the lentivirus family and may add to our understanding of lentivirus evolution.

## Additional files

Additional file 1:
**ELVgv consensus sequence with annotation.** The positions of individual virus genes and their domains were determined by alignments with other lentiviral genomes. In the *env* gene, the position of signal peptide, transmembrane region in TM, and the furin cleavage site between SU and TM subunits were determined by dedicated prediction servers [12-14]. SU, surface glycoprotein; TM, transmembrane glycoprotein; polyA, polyadenylation site. Additional file 2:
**ELVgv consensus sequence.** Sequence identical to Additional file 1, provided in simple text format. Additional file 3:
**(Upper) RNA secondary structure motifs predicted by mfold thermodynamic folding algorithm, with the associated change in Gibbs free energy (dG) [** 10 **].** (Lower) Alignment of homologous regions of the deduced amino acid sequence of ELVgv *orf2* with HIV-1 Tat [GenBank:ABF00629.1]. Additional file 4:
**Alignment of retroviral RT amino acid sequences.** The alignment of 16 retroviral RT amino acid sequences used to generate the tree in Figure 3A. RT domains from the following viruses were used: HIV-1 [GenBank:K03455]; simian immunodeficiency virus, SIV [GenBank:NC758887]; FIV [GenBank:NC001482]; equine infectious anemia virus, EIAV [GenBank:NP_056902]; Visna/maedi virus [GenBank:NC001452]; caprine arthritis encephalitis virus, CAEV [GenBank:NP_040939]; bovine immunodeficiency virus, BIV [GenBank:NP_040563]; Rous sarcoma virus, RSV [GenBank:AF033808]; Mason-Pfizer monkey virus, MPMV[ GenBank:NC001550]; murine leukemia virus, MLV [GenBank:NC001501]; walleye dermal sarcoma virus, WDSV [GenBank:AF033822]; simian foamy virus, SFV [GenBank:NC001364]; bovine leukemia virus, BLV [GenBank:NC_001414]; human T-lyphotropic virus 1, HTLV-1 [GenBank:D13784]; HTLV-2 [GenBank:M10060]. Additional file 5:
**Alignment of lentiviral gag-pol nucelotide sequences.** The alignment of 35 conserved regions of *gag* and *pol* lentiviral sequences from Gilbert *et al*. [5], together with the recently described ELVmpf [6,8] and the ELVgv described here. This alignment was used to generate the tree in Figure 3B. Additional file 6:
**Phylogenetic relationship of ELVgv to other lentiviruses using Bayesian analysis.** The same alignment of 33 lentiviral *gag-pol* sequences as in ML analysis in Figure 3B was used. Bayesian analysis was run for 1,000,000 steps, sampling every 5,000 steps and discarding first 25% of the trees. Average standard deviation of split frequencies converged bellow 0.001. The GTR+I+gamma nucleotide model (a General Time Reversible model with a proportion of invariable sites and a gamma-shaped distribution of rates across sites) was employed in MrBayes program [18]. Appropriate model was selected using program jModeltest vs (Darriba D, et al: jModelTest2: more models, new heuristics and parallel computing. Nat Methods 2012, 9:772). Values of posterior probabilities are shown. Grey lines designate groups of exogenous lentiviruses. The endogenous lentiviruses are denoted by black dots. Scale bar indicates number of nucleotide substitutions per site. Additional file 7:
**Phylogenetic relationship of three full-length ELVgv insertions to other lentiviruses using ML analysis.** The same alignment of lentiviral *gag-pol* sequences as in ML analysis in Figure 3B was used, with the ELVgv consensus sequence substituted by sequences of proviruses *I*, *II* and *III*. The alignment was generated in MEGA5 program [15] using the MUSCLE algorithm [16]. The ML analysis was performed using MEGA5 program under Tamura-Nei model, Nearest-Neighbor-Interchange ML heuristic method and otherwise default parameters. Bootstrap supports (percent out of 1,000 replicates) are shown. Grey lines designate groups of exogenous lentiviruses. The endogenous lentiviruses are denoted by black dots. Scale bars indicate number of nucleotide substitutions per site. Additional file 8:
**List of ELVgv insertions residing in regions of putative genome duplication.** The table lists accession numbers for each pair of colugo genomic contigs that show large regions of apparent segmental duplications (size up to ~ 20 kb). All contigs harbor ELVgv solo LTR sequences in the duplicated regions. The genetic distance of the duplicated region was used to estimate the age of the duplication event, using the same formula as for the estimates based on LTR sequences.
