# Supplementary material for: Endogenous lentivirus in Malayan colugo (Galeopterus variegatus), a close relative of primates
Source: Retrovirology. 2014 Oct 4;11:84. doi: 10.1186/s12977-014-0084-x (PMC4198772; doi:10.1186/s12977-014-0084-x)
Supplement: Additional file 1: — ELVgv consensus sequence with annotation. The positions of individual virus genes and their domains were determined by alignments with other lentiviral genomes. In the env gene, the position of signal peptide, transmembrane region in TM, and the furin cleavage site between SU and TM subunits were determined by dedicated prediction servers [12-14]. SU, surface glycoprotein; TM, transmembrane glycoprotein; polyA, polyadenylation site. [file 12977_2014_84_MOESM1_ESM.pdf]

5' LTR

TGGAGGAAAATGAAGGAGAACACCTGTTAAAGATGGAGGCACTCTTGCTCTCCAGGATTTAGACTGTCAGCAGAAATGCA

AAAAGACTGGGTCAGCAAAAATTCTCAAAGCTTAACCGCAAACCACAGACTTCCTATTGAGTTACCTAGGAGATGGGGAG

TATA box

GTATGGGAGGGGTTTACAGCTGGCTAAAACA**TATATAA**GACATGCTTTTACCCAATAAAGTTGCTTAGTGCATGCGCTAA

GTCCCAGAACTTTGTGTCTGGTTTCTGGTTGCCCACCTCCCCAGGTGGCAGCCCCTAGCTCAAGGCATGCCTTGAGCAAC

TCACCTTCCTGCACTCTCTTGGCAGGGCTGTATGTGGCCCTGCTCCCTGAGGGACTCCTGGCAGAGGCTACGTGTAGTCC

PBS

TGCCTCCTGAGACCCCCAGGACCAAGAGAAATGTGCAGACT**TGGTGCCCAAACATGGGG**CTTGAAGGTTGACCCAAGGAC

TAR hairpin

MA

Gag M

CGGACGGAGGTCTGCAGGACAATGGAGAAGGTGAGTCTGTGCGGCTAGGTCCGGGTAGCAGTAGCTTATAAGCAGG**ATG**G

G T V T S A R L V T S V I Q G I K H C Q S V E K L K G
GGACTGTCACGTCAGCAAGGCTTGTTACTTCAGTCATACAAGGCATTAAGCATTGCCAATCAGTAGAGAAACTGAAGGGA

T G K T T H W A P E D I Y R A F R Q A H V Y C P T F P
ACAGGGAAGACAACACACTGGGCACCTGAGGATATTTATAGGGCATTTCGGCAGGCACATGTTTATTGCCCAACGTTTCC

E T G T L D V R E L R E G I S A L Q K A R K E H P E
TGAAACTGGGACACTAGATGTCAGGGAACTTAGGGAAGGGATAAGTGCCCTTCAGAAAGCAAGAAAAGAGCACCCAGAGT

L S V A V Q A L Q V F L C R G M L G V A V R T T E E V
TGTCTGTAGCTGTGCAAGCCCTGCAGGTATTTCTGTGTAGGGGAATGTTGGGAGTGGCGGTAAGGACCACTGAGGAAGTA

E K V Y F E F R D A G Q I S D S R N I Q G E G K Q V Y
GAAAAGGTATATTTCGAGTTTCGAGATGCTGGCCAGATCTCAGACTCAAGAAATATACAAGGAGAAGGTAAGCAGGTGTA

MA **CA**

P V V Q G P G G T M C S P L H P R V M A D F F D I I
CCCAGTAGTCCAAGGTCCAGGAGGAACTATGTGTTCTCCTCTGCACCCTCGAGTTATGGCAGATTTCTTTGACATCATTA

S H H G L L S S E T I T R M Q V L C M Q D L C Q V E V
GTCATCATGGATTACTGAGCTCAGAAACTATCACTCGAATGCAAGTTTTATGTATGCAGGATTTATGCCAGGTAGAAGTA

K G L I A Q A P G N A A Q K S I L K E L L A K Y K D E
AAAGGCTTGATTGCTCAAGCACCTGGGAATGCTGCACAAAAATCAATTCTAAAAGAATTGTTAGCAAAATATAAAGATGA

W D Q Q N P I Q Q D V F P R Q L T G D K I V G E S P
ATGGGATCAACAAAACCCCATACAGCAAGATGTTTTTCCAAGACAATTGACAGGGGACAAAATAGTAGGAGAATCCCCAA

N N N L P D A Q A G P Q W V I A R S T F Q E W V V N A
ACAATAATTTACCAGATGCACAAGCAGGACCCCAATGGGTAATAGCTAGATCAACCTTCCAGGAGTGGGTTGTCAATGCA

L R E G V K L V Q K P P A V T K I V Q G P R E S Y K D
CTTAGGGAAGGAGTTAAATTAGTTCAGAAACCACCTGCAGTAACAAAAATTGTACAAGGTCCTAGAGAGTCATATAAGGA

F L D R L F E A L E K D F L P L E V S R Y L Q D K L
TTTTTTGGATAGGCTATTTGAAGCATTGGAAAAGGATTTTTTACCTTTAGAAGTTAGTAGATACTTACAGGATAAATTGG

A F E N A N E D C K K M M A H L P P D A A I A D R I H
CTTTTGAAAATGCAAATGAAGATTGTAAAAAGATGATGGCTCACTTGCCTCCGGATGCAGCAATTGCTGACAGGATACAT

CA **NC**

C C R S V G T V T H K Q T M M A E A F A A A L F K G K
TGCTGCAGATCCGTGGGTACAGTGACCCACAAACAAACCATGATGGCAGAGGCTTTTGCAGCAGCTCTCTTTAAAGGAAA

G N Q Q G L R C F N C N K L G H M K K D C K Q Q R K
AGGGAACCAGCAAGGGCTTAGATGTTTTAATTGCAATAAACTAGGCCATATGAAAAAAGATTGTAAGCAGCAAAGAAAGG

PR

Pol K G T H C Q V M Q N K K Q

A T A N K D V T C F K C G K K G H I A R L C R T K N R
CTACAGCTAATAAAGATGTTACATGTTTCAAATGTGG**AAAAAA**GGGACACATTGCCAGGTT**ATG**CAGAACAAAAAACAGG

slippery sequence frame-shift hairpin

E S R L R T G S P G I G I T A C S A I S T S G G R D N
K A G S G Q G A Q A L V S Q P V L P S A P V E E E T T
AAAGCAGGCTCAGGACAGGGAGCCCAGGCATTGGTATCACAGCCTGTTCTGCCATCAGCACCAGTGGAGGAAGAGACAAC

NC

Q V T L P Q I T S L Q Q R P I T L I Q C A G T E I K V
K S L Y P K L P V C N K D Q *
CAAGTCACTTTACCCCAAATTACCAGTTTGCAACAAAGACCAA**TAA**CATTGATACAGTGTGCAGGGACAGAGATTAAGGT

L L D T G A D I S I W A K P P P I G R N I G Q Q F V
TTTATTAGATACAGGAGCAGATATTTCAATTTGGGCAAAACCACCCCCAATAGGAAGAAATATAGGCCAGCAATTTGTAA

M G I G G S Q T G I K Y D Q V K M T W N N K T I L G E
TGGGAATAGGGGGAAGTCAAACAGGAATTAAATATGATCAAGTTAAAATGACATGGAATAATAAAACAATTTTAGGTGAA

V V V C P T P I N L L G R D N L S K F G V H L V C T T
GTAGTAGTGTGTCCCACACCTATAAATTTATTAGGGAGAGATAATTTAAGCAAATTTGGAGTACACTTGGTGTGTACAAC

PR **RT**

L N Q Q L T P V K F K L L P G F R G P Q V K Q W P L
GTTAAATCAACAGTTAACACCAGTGAAATTTAAATTGTTGCCAGGATTTAGAGGACCACAGGTTAAGCAATGGCCATTAT

S E E K I K A L Q E I C A E L E Q E K K I Q R V G P E
CTGAAGAAAAAATTAAAGCATTACAAGAAATTTGTGCTGAATTAGAACAGGAAAAAAAGATACAAAGAGTAGGTCCAGAA

N P Y N T P V F L I K K K S G K W R M L M D F R E L N
AATCCTTATAATACACCTGTATTTTTAATAAAAAAGAAATCTGGTAAGTGGAGGATGCTTATGGATTTTAGGGAATTAAA

K G I D V G T E T Q V G F P H P A G F S Q V K H F T
CAAAGGAATAGATGTAGGAACAGAAACTCAGGTAGGTTTTCCTCATCCAGCAGGGTTTTCACAGGTAAAGCACTTTACTG

V I D I K D A Y F T I P L D E E I S E L T A F T V P Q
TTATTGATATAAAAGATGCTTATTTCACCATCCCATTAGATGAAGAAATAAGTGAGTTAACAGCATTTACAGTACCTCAA

K N N A G P G I R F K W K V L P Q G L I G S P K G Y Q
AAGAATAATGCAGGGCCAGGTATAAGATTTAAATGGAAAGTTTTACCACAAGGGTTGATAGGGTCTCCAAAAGGGTATCA

C T L E K I I T P W K A K H P G I T C Y V Y M D D I
ATGTACTTTAGAAAAAATAATAACACCATGGAAAGCAAAACACCCTGGCATAACATGTTATGTATATATGGATGATATTT

L I G T N Y T A L K Y K Q V V K E L Q E E F K K W G F
TAATAGGAACAAATTATACAGCTCTCAAATATAAACAAGTAGTCAAAGAGTTACAAGAGGAATTTAAAAAGTGGGGATTT

E T P Q D K I Q N S D G D K A V K Y L G Y I L E P Q T
GAAACCCCACAAGATAAAATACAGAATAGTGATGGGGACAAAGCAGTAAAATATTTAGGATATATTTTAGAACCCCAAAC

W K P H S Y Q L L D S K P K T L N N V Q R V L G R I
GTGGAAACCACATAGCTATCAGCTATTGGATAGTAAACCAAAAACATTAAATAATGTTCAACGAGTATTAGGAAGAATTA

N W I K Q I Y P K I Q T E N I Q K L L T G D Q N I L S
ATTGGATAAAACAAATATATCCTAAGATACAAACAGAAAACATTCAGAAGTTATTAACAGGAGATCAAAACATATTAAGT

K R S W T K K T Q Y E V D V I Q Q M L T Q Q E G M P Y
AAAAGATCATGGACTAAAAAAACTCAATATGAGGTAGATGTAATACAACAAATGTTAACACAGCAGGAAGGAATGCCTTA

H Q E K E L V S L S I V W N Q V A Q G A V H Q K H K
TCATCAAGAAAAGGAACTAGTTAGTCTTAGTATTGTGTGGAATCAAGTAGCTCAAGGAGCTGTCCATCAAAAGCATAAGG

V L W W G Y G T S K D R K I K T K A R K C V A L A N R
TGCTCTGGTGGGGATACGGGACTAGCAAAGACAGAAAAATAAAAACTAAAGCAAGAAAATGTGTGGCTTTAGCAAATCGT

M V S E I I I L L G K V P D E V W L H I E K A T F E Y
ATGGTTTCAGAAATAATCATACTATTGGGAAAAGTTCCAGATGAAGTTTGGTTGCACATAGAAAAGGCTACTTTTGAGTA

L L Q E G L E G T E I W L L N C K F G M A P N K I F
TTTATTACAAGAAGGATTAGAGGGAACAGAAATTTGGTTATTAAATTGCAAATTTGGTATGGCACCTAACAAAATTTTTG

RT **RNase H**

D K S E C L S Y P C I W K S A V P V T G P T I Y I D G
ATAAAAGTGAATGTTTAAGTTATCCTTGTATATGGAAAAGTGCTGTTCCTGTCACAGGCCCTACAATTTATATAGATGGA

S R K K G Q P A R S A I W E N A N N N K V T E F E G T
TCTAGAAAAAAGGGACAGCCAGCTCGTAGTGCTATATGGGAGAATGCAAATAATAATAAAGTAACAGAATTTGAGGGAAC

A Q Q A E V R A F L L A L Q L E Y N Q M N I V T D S
AGCTCAGCAAGCAGAAGTTAGGGCATTTTTATTAGCATTACAATTAGAATATAATCAAATGAACATAGTTACAGACAGTA

K Y L W T C I K L M S D Y A W K E E N P I W Q E I W E
AATATTTATGGACATGTATTAAGTTAATGTCTGACTATGCGTGGAAAGAGGAAAATCCCATTTGGCAAGAAATCTGGGAA

RNaseH

L L Q T K K I Y L Q W V P G H Q G V P G N E E A D N L
TTATTACAAACAAAGAAAATATATTTACAATGGGTGCCAGGACATCAAGGGGTGCCAGGGAATGAAGAAGCTGACAATTT

dUTPase

A Q G N V V L T A E E E D L L P Q Q S E L C F P G W
AGCCCAGGGAAATGTAGTTCTTACTGCAGAAGAGGAGGACTTACTGCCACAGCAGTCAGAACTTTGTTTCCCTGGGTGGC

Q I K A A R D C Y V A A Q E K R Q I F T G I H L Q L D
AAATAAAAGCAGCCAGAGATTGTTATGTGGCAGCCCAGGAAAAAAGACAAATATTTACAGGAATACACCTCCAGTTGGAT

E M Q I A Q M Q L N Y E L A I K G L L L L Q S T V K E
GAAATGCAAATAGCACAGATGCAATTAAATTATGAATTAGCTATAAAAGGATTGTTATTATTACAGTCAACTGTTAAAGA

S Y S T E L I L Q V V N F T K K G I N I Q K G D P V
AAGTTATTCAACAGAATTAATATTACAAGTTGTAAATTTTACTAAAAAGGGAATAAATATTCAAAAGGGTGATCCAGTTG

dUTPase

V I L Y I I A A E E M G E A K L Q K S A P D D M A C F
TTATTTTATATATTATAGCTGCTGAAGAGATGGGGGAAGCAAAGTTACAAAAGTCAGCTCCAGATGACATGGCCTGTTTC

INT

T S E T F Q E A E Q W H D V T H A N P R Q L A R K F H
ACCTCAGAAACTTTTCAGGAAGCAGAACAGTGGCATGATGTAACACATGCAAACCCCCGACAATTAGCAAGAAAGTTTCA

L P L G L A K H I T Q L C K D C Q Q Q D T A T G E G
TCTTCCTTTAGGATTAGCAAAGCATATTACACAGTTATGCAAAGACTGCCAACAGCAGGATACTGCTACAGGAGAGGGAT

Y N Q L K N Q E G V W Q M D V T H M F E L G L Y K Y V
ATAATCAGTTAAAAAACCAAGAAGGTGTTTGGCAGATGGATGTTACACATATGTTTGAGTTAGGACTTTATAAATATGTG

Y V A V D I Q T G M I W V S P Q K G E T A K H T Q T A
TATGTAGCAGTTGACATACAAACAGGAATGATATGGGTTAGTCCACAAAAGGGAGAAACAGCAAAACATACACAAACAGC

L L Q I I H M A G L P K E I Q S D N G P G F V A D R
TCTGCTTCAGATCATTCATATGGCAGGGCTCCCAAAGGAAATCCAAAGTGATAATGGACCAGGTTTTGTAGCAGATAGAG

V Q V M C K Q L G I K W H H G I P Y H P Q S Q G K V E
TACAAGTGATGTGTAAACAATTAGGGATTAAATGGCATCATGGCATTCCATACCATCCTCAGAGTCAGGGAAAAGTAGAA

G T H K L I K Q H F K K V K E L F E D P I N A L L W T
GGTACACATAAATTAATTAAACAACACTTCAAAAAGGTGAAAGAGTTATTTGAAGACCCCATTAATGCTCTGTTGTGGAC

V F C L N F E K R G G D G Y T R A E A W V E Q Q N F
AGTATTTTGCCTCAATTTTGAGAAGAGGGGTGGAGATGGGTATACAAGAGCAGAGGCTTGGGTGGAGCAACAGAATTTTA

K N Q L E T C K H M Q T L T N S C K F L Y K D P K S P
AAAATCAGTTAGAAACATGTAAGCATATGCAAACTTTAACTAATTCCTGTAAATTTTTGTATAAGGATCCCAAGTCACCT

R T G W R G P G T L Q W E G A G I R V L E L T T G E V
CGAACAGGGTGGAGAGGCCCAGGCACTCTGCAGTGGGAAGGAGCAGGCATCAGAGTATTAGAACTTACAACTGGAGAGGT

INT

ORF1 M K P R V Q M N P E V T V

K V V P K H L T K R I F S D E A *
AAAAGTAGTTCCTAAACATCTAACAAAAAGAATTTTTTCAG**ATG**AAGCC**TAG**AGTTCAGATGAATCCTGAAGTTACTGTT

Q W M D A F C F G Q E E A I I I A Y I I N G Q E L H P
CAGTGGATGGATGCCTTTTGTTTTGGACAAGAAGAAGCCATTATTATAGCTTACATCATTAATGGCCAAGAGTTACACCC

G E L M V V H K Q T I P R W L T Q V T R S M W N K H
TGGTGAACTTATGGTAGTGCATAAGCAAACTATTCCTAGATGGTTAACACAGGTTACTCGTTCTATGTGGAATAAACATA

R T S T E Q R L I Q I Q K R Y W I N T F C G P K R Y L
GAACAAGTacagaACAGAGATTAATCCAAATACAAAAAAGATATTGGATCAATACATTTTGTGGACCTAAAAGATAtCtC

L V K G W E Q K V K W V K H I Q L L P K F G N I C M W
TTAGTGAAAGGATGGGAACAAAAGGTAAAATGGGTCAAACACATACAGTTGTTGCCAAAGTTTGGGAACATATGTATGTG

C P G V K V P H W S E P Y G A P K P R G S Y L V Q L
GTGCCCTGGTGTTAAAGTTCCCCATTGGAGTGAACCATATGGTGCTCCAAAGCCTCGTGGATCTTATTTAGTGCAGTTAA

R Q L I N H M V E E T H P S P R G T C G W P Y R C P P

GACAATTAATAAATCATATGGTAGAAGAAACTCATCCTAGCCCCAGAGGAACTTGTGGATGGCCTTACAGATGCCCACCA

C V L Q A A I I L L E E D Y W K D E E V I P K L Q M I

TGTGTTCTTCAAGCAGCTATTATATTACTAGAGGAGGATTATTGGAAGGAtGAGGAAGTTATTCCAAAGTTACAAATGAT

C Y S R C L K Q L W K E M T Y Q I S K R N I S T W H

ATGTTATAGTAGATGTTTAAAACAATTGTGGAAAGAAATGACTTATCAAATTTCAAAAAGAAATATTAGTACATGGCATG

A R M Q T P G S T K W Q S P K H G P H V I T G I P W Q

CTAGGATGCAAACACCAGGAAGTACAAAATGGCAATCACCAAAACATGGCCCTCATGTCATTACAGGAATCCCTTGGCAA

R L A T K F C V W E A N G D L K S E A * **ORF2 (Tat)** M

AGACTGGCAACAAAATTTTGTGTTTGGGAGGCTAACGGGGATTTAAAATCAGAAGCA**TAA**AGAGTAACCCATGGTAA**ATG**

C C E C R C L C L H C C Y H C I L C F Q Q K Y L G I T

TGTTGTGAATGTAGATGCCTTTGCTTGCACTGCTGTTATCATTGCATTTTATGCTTTCAGCAAAAATACTTAGGGATAAC

H E G G P R G I K P C C H K C Y K P K Q G G K Y C G

ACATGAAGGAGGTCCCAGAGGAATAAAACCTTGTTGTCATAAATGTTATAAGCCAAAACAGGGAGGTAAATACTGTGGGA

T K N S F E V H L Q P C F H K C C R R P I S S G R K R

CAAAAAACAGTTTTGAAGTACATTTACAACCTTGTTTTCATAAaTGTTGTAGACGACCTATTTCAAGTGGAAGGAAAAGA

K Y N T G P K Q G S K S S G T R N N S I L N *

AAGTACAATACTGGTCCAAAACAAGGGAGCAAATCCTCTGGGACAAGGAACAATTCTATATTAAAT**TAA**GAATACAGATC

CCAAGAACACTTAGTGAAGGAAGGGAAAATAATCCTCAGCTGCTACCCTCGTTGTTGCAAAAGGAATTGTGAGCAACAGT

TAGATCCAGTCCCTGAAgGAAACCATGGCCTCTTCCTCCTTACCATTAATAAATAATGAGGAAGATGATTTTTATATGGT

AATGACTCCTAGATTGTTAACAAAAGGAACTCAAACTGACCAAATAAAAATGAGATCCCAAAAGGCAGGACCATCCAAGG

AGGATAAAATAATGGAAGAATTAAGGGTGCTTCAGCTTCAACAGGAGAAAGAGGAGAGATGGAGACAACAAGAGCTGTGT

SU

Env M P L L P A G K G K N I I W V

ACCACCAGCTCATCTGgAGAGGACAAGGACGTGAGT**ATG**CCTTTGTTACCGGCAGGAAAAGGTAAGAATATTATATGGGT

signal peptide

C I L A H L W V P T C Q G F R L G L L K W S S A S T

CTGTATTTTAGCACATTTGTGGGTCCCCACATGTCAAGGTTTTAGGCTAGGGTTGTTAAAATGGTCCAGTGCATCTACAC

L V S P Q K V P W F C A A A N T Q F G C L P K N H Y I

TTGTAAGTCCTCAAAAAGTCCCTTGGTTTTGTGCAGCAGCCAATACCCAATTTGGATGTCTTCCCAAAAACCATTACATA

T Q L H W A G N N T H M W G L R G H R N G T E Y G N N

ACACAGCTGCATTGGGCAGGAAACAATACCCATATGTGGGGACTTAGAGGACACAGAAATGGTACAGAATATGGAAACAA

N W G L Q I G K Q W V W L F E K Y L N T Q C P K N T

TAATTGGGGGCTTCAAATAGGAAAACAATGGGTATGGTTATTTGAAAAATATTTAAATACTCAATGTCCAAAAAATACAG

A Y C K P F N C T N A T W P L N V T E D Y W G A N C S

CTTATTGTAAGCCATTTAATTGTACAAATGCCACCTGGCCTTTAAATGTAACTGAGGATTATTGGGGAGCAAATTGTAGT

G L E W I S D S T G R I F Q G H K P M I F N T T W A L

GGACTTGAATGGATTTCAGATTCCACTGGCAGGATATTTCAAGGACATAAGCCTATGATCTTTAATACCACTTGGGCATT

K Q G L V N Q I S C K P N H T A Q G S Q C K K S N K

AAAGCAAGGACTTGTAAATCAAATCAGTTGTAAACCAAATCATACTGCTCAAGGGTCTCAGTGTAAGAAAAGCAATAAAG

A W C C N W K D C N Q F Q D I L E A C P E K L R L L T

CTTGGTGTTGTAATTGGAAAGATTGCAATCAATTTCAAGATATTTTAGAAGCATGCCCTGAAAAGCTCAGGTTGCTCACT

S F T K G V Q L C V H R P Y Y F W S C N K H K I Q R E

TCTTTTACCAAAGGAGTACAGTTATGTGTCCATAGACCTTATTATTTTTGGAGTTGTAACAAACACAAAATTCAAAGAGA

Y D T N S N V S G E F K W G N E T I V G S S T N R Q

GTATGACACAAACAGTAATGTTAGTGGTGAATTTAAATGGGGGAATGAAACAATTGTGGGCAGTAGTACCAACAGACAAA

N E T L Q L V K W E C D G E A Q V T Q C L P E L G E L

ATGAAACTTTACAGCTAGTAAAATGGGAATGTGACGGAGAAGCTCAAGTCACTCAGTGCTTACCCGAGTTAGGTGAACTT

T A D S T F I G I G W K N W I Y P Q L V W Q A K F V N
ACAGCTGATAGTACATTTATAGGAATAGGATGGAAAAACTGGATATATCCTCAATTGGTCTGGCAGGCCAAATTTGTAAA

L T N N N T W R I P T S E T N D Q S I K T E Q S W L
TCTGACAAATAATAATACATGGAGAATTCCAACAAGTGAAACAAATGACCAAAGCATCAAAACTGAACAGTCCTGGCTAC

H S I K F N K T H P G Q V N C T I M H L S S Y T N K K
ATAGTATAAAATTTAATAAAACTCATCCAGGACAAGTAAATTGTACTATTATGCATTTATCTAGTTATACTAATAAGAAG

Q Y M P F N S L A F C N L S L P G Q V M K R C L S N I
CAATACATGCCTTTTAATAGCTTAGCATTTTGCAATTTATCCTTGCCAGGGCAAGTCATGAAAAGGTGTTTATCAAACAT

S N L P E E G K F D C A I P H P W V A G D F Q R W T
TAGTAATTTGCCAGAGGAAGGGAAATTTGATTGTGCTATCCCACATCCTTGGGTAGCAGGGGATTTCCAAAGATGGACTT

W Q Y T K R Q G D D E P S T P H L S F P C E M K D K Q
GGCAATATACAAAAAGGCAAGGGGATGATGAGCCATCAACACCACATTTGTCCTTTCCATGTGAGATGAAAGACAAACAA

L F Q L E C N F V N K T K N A N D T Q F C Q D C L E C
TTATTTCAATTAGAGTGCAATTTTGTTAATAAAACTAAAAATGCAAATGATACTCAATTTTGTCAAGATTGTTTAGAATG

R S R D T R I L L W T H K L M H V R S S G E Y K Y T
TAGATCCAGGGATACTAGGATCTTACTATGGACTCACAAATTAATGCATGTACGGTCATCAGGGGAATACAAATATACAA

T C S F Q F I G I H C D S L R V K G Q E G T Q W K K F
CATGTAGTTTCCAATTTATAGGAATTCATTGTGACTCATTAAGAGTTAAAGGCCAGGAAGGCACACAATGGAAAAAATTT

D I E S S Q C D M L F N V S A P E G A A G T P N S T C
GACAtaGAAAGTAGTCAGTGTGACATGTTGTTCAATGTATCAGCACCAGAGGGAGCAGCTGGCACACCAAACAGTACATG

A R S W S A I C F A T T G G P E E W I Q Q A F D R H
TGCTCGTTCATGGTCAGCCATTTGTTTTGCAACTACAGGAGGCCCAGAGGAATGGATTCAACAGGCTTTTGACAGACATA

I K V G R Q E N I I P I P I D D N K L E I V K P K R S
TTAAGGTGGGCCGACAGGAAAATATAATTCCCATTCCTATAGATGATAACAAGTTAGAAATTGTTAAGCCAAAGAGATCC

SU ← → **TM**

K R | G A V L L A A F G I V T A I T A V A G M V A G G V
AAGCGTGGAGCTGTTTTATTGGCTGCTTTCGGAATTGTTACTGCCATCACTGCTGTGGCTGGTATGGTTGCAGGTGGGGT

fusion peptide

A L H Q L Q E L K E L A D K N V Q M M V D L I K T Q
GGCCCTCCATCAATTACAGGAACTCAAGGAACTAGCAGACAAAAATGTCCAGATGATGGTGGATCTGATAAAGACCCAGG

E N H W V F T R D L T F G L L E L E A R V C W I E E V
AAAATCATTGGGTTTTTACAAGGGATCTTACATTTGGACTTTTGGAGCTAGAAGCTCGAGTATGCTGGATTGAGGAGGTG

L A T D K A F Q T L N C E I S A G S L C I F R E R Y N
CTGGCCACTGACAAAGCCTTTCAAACATTAAACTGTGAAATATCAGCTGGCAGTCTTTGCATTTTCCGAGAGCGGTACAA

T T Q L K E W I N S T S W Y N M S I N E W A A R L Q
TACAACACAGCTCAAAGAATGGATCAATAGCACCAGCTGGTACAACATGTCAATTAATGAATGGGCAGCAAGATTACAGT

F F Q K G I K E I D K L V L K N R V G L T H L R H S L
TTTTTCAAAAGGGCATAAAAGAAATAGATAAATTAGTTCTTAAAAATAGAGTAGGTTTAACACACTTAAGACATAGTTTA

Q K V Q E Q S T L E A W I S T V P D W L S S I W R A L
CAGAAGGTACAAGAACAGTCAACATTAGAGGCATGGATTAGTACAGTGCCTGATTGGTTGTCTTCCATATGGAGGGCATT

K K S W W W L I I G I I C L V I I I P L L P S C I K
AAAAAAGAGTTGGTGGTGGCTCATCATAGGCATAATTTGTTTGGTCATCATTATTCCCTTGTTACCAAGTTGTATTAAAG

transmembrane region

TM

ORF3 I C L I Y R N N G H E R D D G

A I I N Y I R G Y V I V N M F D I Q E Q W P R E R *
CAATCATAAATTATATCAGAGGATATGTAATAGTAAATATGTTTGATATACAGGAACAATGGCCACGAGAGAGA**TGA**TGG

Q Q D K K T Q E K K T Q T K G K T K S N N T C P N T
ACAACAAGATAAAAAGACACAAGAAAAGAAAACTCAAACAAAAGGAAAAACAAAGAGCAACAACACTTGCCCAAACACCA

I P F A F R L K S R C C S T W I S S Y S G N R K R D L
TCCCCTTCGCTTTTAGGCTTAAAAGCCGCTGCTGCAGCACTTGGATATCATCTTATTCCGGCAATAGAAAAAGAGATTTA

```
 N   Q   N   H   A   I   V   A   V   T   V   E   A   A   Y   *
AACCAAAATCATGCTATTGTTGCTGTTACTGTTGAGGCTGCATATTAATCAGATGGCTTTTTATTGTGTGTATTTAGACT
                                             5' LTR
TTGCCAACAATTGCAAGCCTGCTTTTAAAAGAAAAAAGGGTGGACTGGAGGAAAATGAAGGAGAACACCTGTTAAAGATG
                               PPT
GAGGCACTCTTGCTCTCCAGGATTTAGACTGTCAGCAGAAATGCAAAAAGACTGGGTCAGCAAAAATTCTCAAAGCTTA

ACCGCAAACCACAGACTTCCTATTGAGTTACCTAGGAGATGGGGAGGTATGGGAGGGGTTTACAGCTGGCTAAAACATAT
                    polyA
ATAAGACATGCTTTTACCCAATAAAGTTGCTTAGTGCATGCGCTAAGTCCCAGAACTTTGTGTCTGGTTTCTGGTTGCCC

ACCTCCCCAGGTGGCAGCCCCTAGCTCAAGGCATGCCTTGAGCAACTCACCTTCCTGCACTCTCTTGGCAGGGCTGTATG

TGGCCCTGCTCCCTGAGGGACTCCTGGCAGAGGCTACGTGTAGTCCTGCCTCCTGAGACCCCCAGGACCAAGAGAAATGT

GCA
```

Legend

PBS - Primer binding site
Tat - Trans-activator of transcription
TAR - Trans-activation response element
MA - Matrix
CA - Capsid
NC - Nucleocapsid
PR - Protease
RT - Reverse transcriptase
INT - Integrase
SU - Envelope surface unit protein
TM - Envelope transmembrane protein
PPT - Polypurine tract
polyA - Polyadenylation signal site
