# Supplementary material for: Endogenous lentivirus in Malayan colugo (Galeopterus variegatus), a close relative of primates
Source: Retrovirology. 2014 Oct 4;11:84. doi: 10.1186/s12977-014-0084-x (PMC4198772; doi:10.1186/s12977-014-0084-x)
Supplement: Additional file 3: — (Upper) RNA secondary structure motifs predicted by mfold thermodynamic folding algorithm, with the associated change in Gibbs free energy (dG) [ 10 ]. (Lower) Alignment of homologous regions of the deduced amino acid sequence of ELVgv orf2 with HIV-1 Tat [GenBank:ABF00629.1]. [file 12977_2014_84_MOESM3_ESM.pdf]

Additional file 3

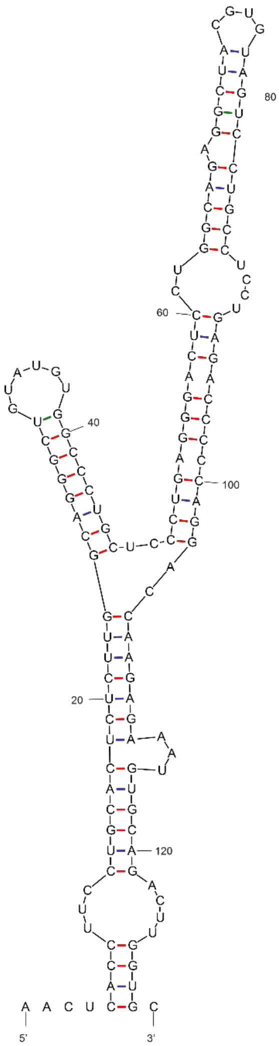

TAR
dG = -67.00

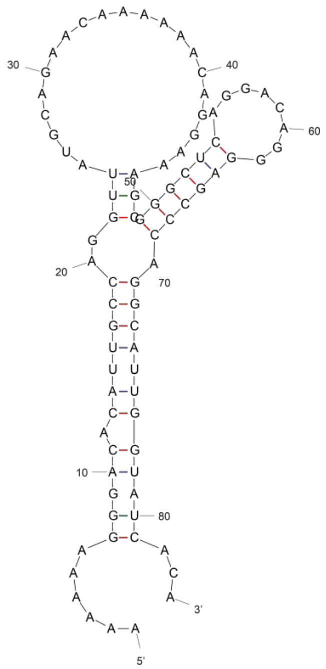

Gag-pol frame-shift hairpin
dG = -17.70

```
ELVgv ORF2     7  CLCLHCCYHCILCFQQKYLGITH 29
                  | | ||||||..|||.| |||..
HIV-1 Tat     25  CFCKHCCYHCLVCFQKKGLGISY 47
```
