# Supplementary figures and images for: Endogenous lentivirus in Malayan colugo (Galeopterus variegatus), a close relative of primates
Source: Retrovirology. 2014 Oct 4;11:84. doi: 10.1186/s12977-014-0084-x (PMC4198772; doi:10.1186/s12977-014-0084-x)

## Additional file 6

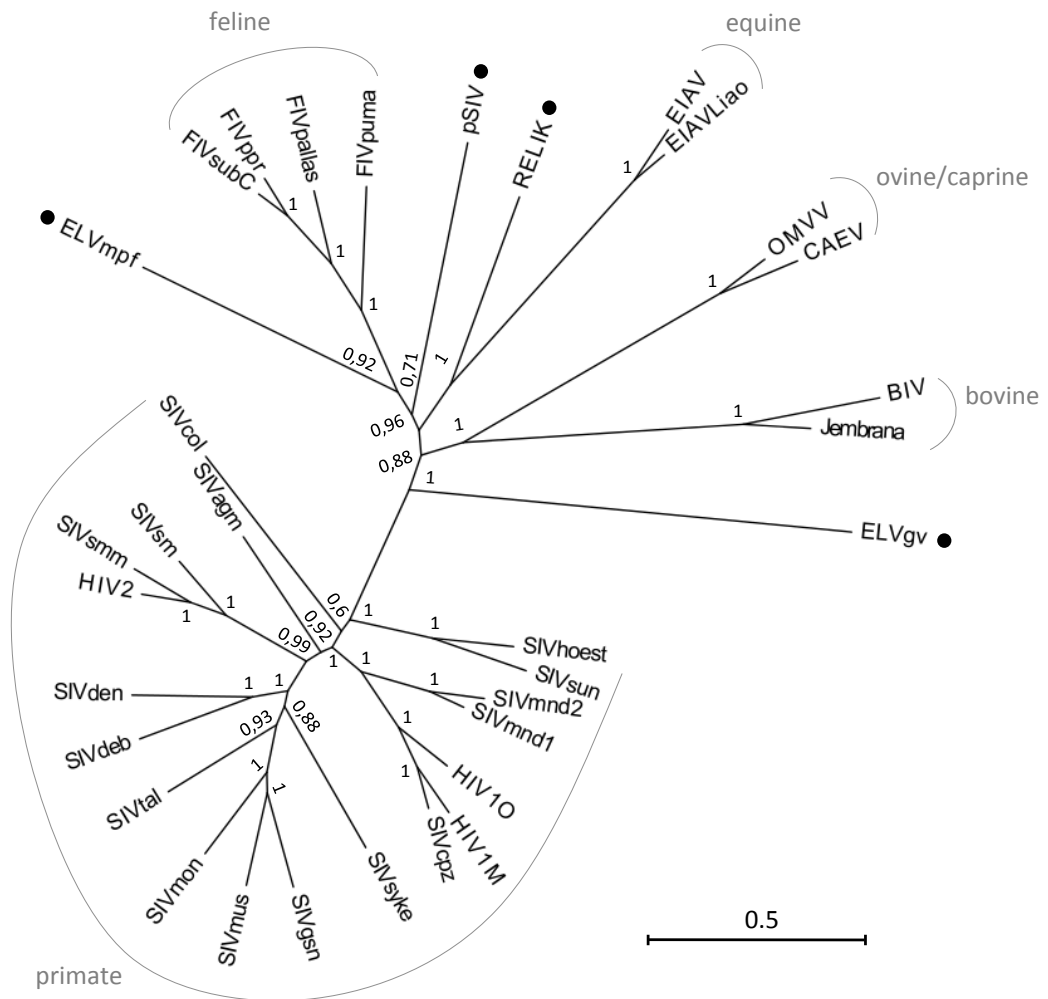

Supplement: Additional file 6: — Phylogenetic relationship of ELVgv to other lentiviruses using Bayesian analysis. The same alignment of 33 lentiviral gag-pol sequences as in ML analysis in Figure 3B was used. Bayesian analysis was run for 1,000,000 steps, sampling every 5,000 steps and discarding first 25% of the trees. Average standard deviation of split frequencies converged bellow 0.001. The GTR+I+gamma nucleotide model (a General Time Reversible model with a proportion of invariable sites and a gamma-shaped distribution of rates across sites) was employed in MrBayes program [18]. Appropriate model was selected using program jModeltest vs (Darriba D, et al: jModelTest2: more models, new heuristics and parallel computing. Nat Methods 2012, 9:772). Values of posterior probabilities are shown. Grey lines designate groups of exogenous lentiviruses. The endogenous lentiviruses are denoted by black dots. Scale bar indicates number of nucleotide substitutions per site. [file 12977_2014_84_MOESM6_ESM.pdf]

Additional file 7

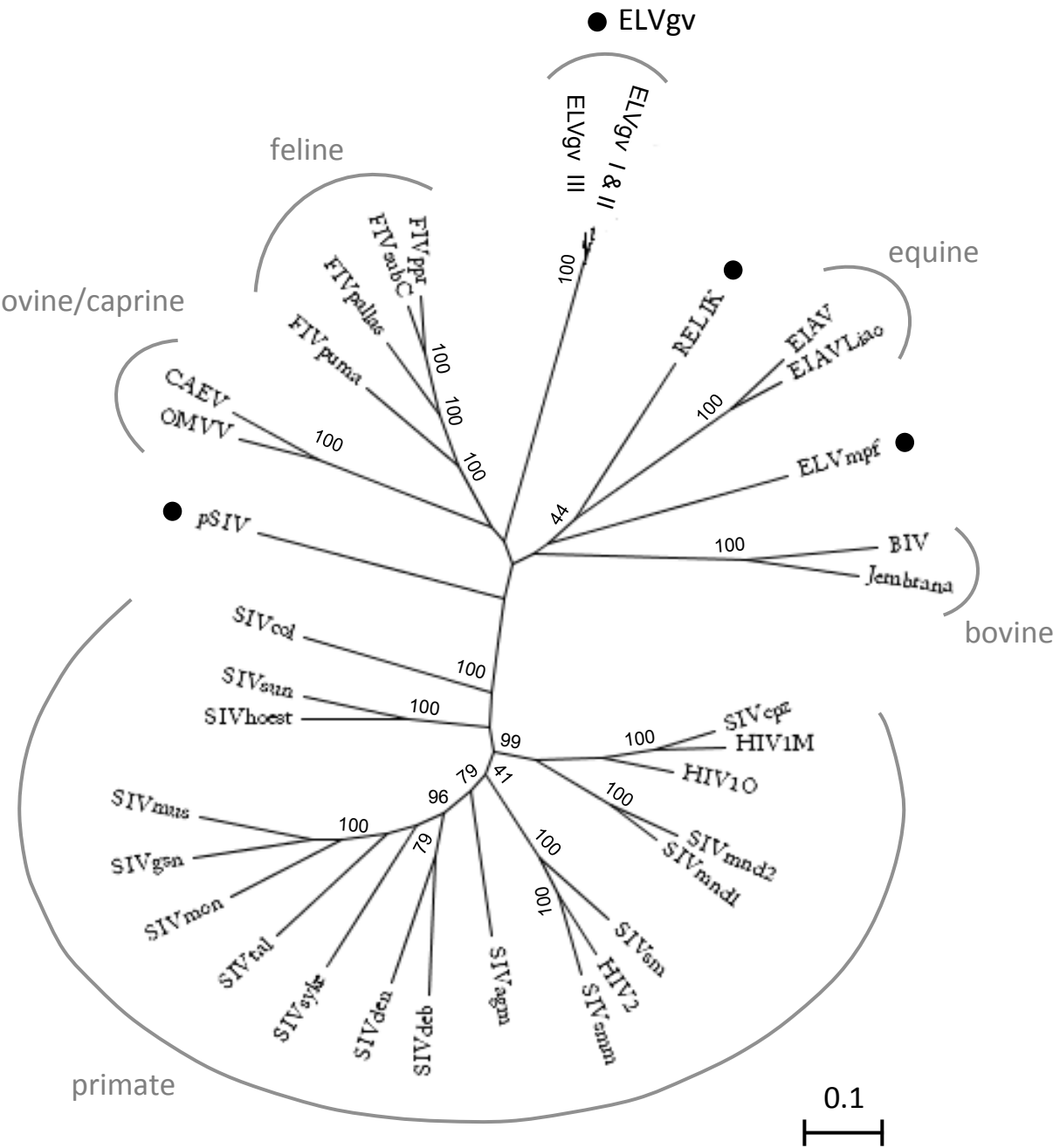

Supplement: Additional file 7: — Phylogenetic relationship of three full-length ELVgv insertions to other lentiviruses using ML analysis. The same alignment of lentiviral gag-pol sequences as in ML analysis in Figure 3B was used, with the ELVgv consensus sequence substituted by sequences of proviruses I, II and III. The alignment was generated in MEGA5 program [15] using the MUSCLE algorithm [16]. The ML analysis was performed using MEGA5 program under Tamura-Nei model, Nearest-Neighbor-Interchange ML heuristic method and otherwise default parameters. Bootstrap supports (percent out of 1,000 replicates) are shown. Grey lines designate groups of exogenous lentiviruses. The endogenous lentiviruses are denoted by black dots. Scale bars indicate number of nucleotide substitutions per site. [file 12977_2014_84_MOESM7_ESM.pdf]
