# Supplementary material for: Endogenous lentivirus in Malayan colugo (Galeopterus variegatus), a close relative of primates
Source: Retrovirology. 2014 Oct 4;11:84. doi: 10.1186/s12977-014-0084-x (PMC4198772; doi:10.1186/s12977-014-0084-x)
Supplement: Additional file 8: — List of ELVgv insertions residing in regions of putative genome duplication. The table lists accession numbers for each pair of colugo genomic contigs that show large regions of apparent segmental duplications (size up to ~ 20 kb). All contigs harbor ELVgv solo LTR sequences in the duplicated regions. The genetic distance of the duplicated region was used to estimate the age of the duplication event, using the same formula as for the estimates based on LTR sequences. [file 12977_2014_84_MOESM8_ESM.docx]

| **contig 1**  **[accession number]** | **contig 2**  **[accession number]** | **distance of putative duplicated regions** | **calculated age [millions years ago]** |
| --- | --- | --- | --- |
| 639890467 | 647074667 | 0.003 | 0.33-0.68 |
| 647024172 | 647024172 | 0.023 | 2.56-5.23 |
| 647029750 | 647068172 | 0 | 0.00 |
| 647063778 | 647066827 | 0.032 | 3.56-7.27 |
| 639651268 | 646997675 | 0.024 | 2.67-5.45 |
| 646887812 | 647013347 | 0.008 | 0.89-1.82 |
| 639729983 | 646997675 | 0.025 | 2.78-5.68 |
